# Bayesian Distance Clustering

**Published:** 2021

**Authors:** Leo L Duan, David B Dunson

**Affiliations:** Department of Statistics, University of Florida, Gainesville, FL 32611, USA; Department of Statistical Science, Duke University, Durham, NC 27708, USA

**Keywords:** Distance-based clustering, Mixture model, Model-based clustering, Model misspecification, Pairwise distance matrix, Partial likelihood

## Abstract

Model-based clustering is widely used in a variety of application areas. However, fundamental concerns remain about robustness. In particular, results can be sensitive to the choice of kernel representing the within-cluster data density. Leveraging on properties of pairwise differences between data points, we propose a class of Bayesian distance clustering methods, which rely on modeling the likelihood of the pairwise distances in place of the original data. Although some information in the data is discarded, we gain substantial robustness to modeling assumptions. The proposed approach represents an appealing middle ground between distance- and model-based clustering, drawing advantages from each of these canonical approaches. We illustrate dramatic gains in the ability to infer clusters that are not well represented by the usual choices of kernel. A simulation study is included to assess performance relative to competitors, and we apply the approach to clustering of brain genome expression data.

## 1. Introduction

Clustering is a primary focus of many statistical analyses, providing a valuable tool for exploratory data analysis and simplification of complex data. In the literature, there are two primary approaches – distance- and model-based clustering. Let $y_{i}\in Y$, for *i* = 1, …, *n*, denote the data and let *d*(*y*, *y*′) denote a distance between data points *y* and *y′*. Then, distance-based clustering algorithms are typically applied to the *n* × *n* matrix of pairwise distances *D*_(*n*)×(*n*)_ = {*d*_*i,j*_}, with *d*_*i,j*_ = *d*(*y*_*i*_, *y*_*j*_) for all *i*, *j* pair and (*n*) = {1, …, *n*}. For reviews, see Jain (2010); Xu and Tian (2015). In contrast, model-based clustering takes a likelihood-based approach in building a model for the original data *y*_(*n*)_ that has the form:

(1) $$y_{i}\overset{\text{iid}}{\sim }f,\;f(y)=\sum_{h=1}^{k}\pi_{h}K\left(y;\theta_{h}\right),$$

where *π* = (*π*_1_, …, *π*_*k*_)′ is a vector of probability weights in a finite mixture model, *h* is a cluster index, and $K\left(y;\theta_{h}\right)$ is the density of the data within cluster *h*. Typically, K(y;θ) is a density in a parametric family, such as the Gaussian, with *θ* denoting the parameters. The finite mixture model (1) can be obtained by marginalizing out the cluster index *c*_*i*_ ∈ {1, …, *k*} in the following model:

(2) $$y_{i}\sim K\left(\theta_{c_{i}}\right),\text{ pr}\left(c_{i}=h\right)=\pi_{h}.$$

Using this data-augmented form, one can obtain maximum likelihood estimates of the model parameters *π* and *θ* = {*θ*_*h*_} via an expectation-maximization algorithm (Fraley and Raftery, 2002). Alternatively, Bayesian methods are widely used to include prior information and characterize uncertainty in the parameters. For reviews, see Bouveyron and Brunet-Saumard (2014) and McNicholas (2016).

Distance-based algorithms tend to have the advantage of being relatively simple conceptually and computationally, while a key concern is the lack of characterization of uncertainty in clustering estimates and associated inferences. While model-based methods can address these concerns by exploiting a likelihood-based framework, a key disadvantage is a large sensitivity to the choice of kernel K(⋅;θ). Often, kernels are chosen for simplicity and computational convenience, and they place rigid assumptions on the shape of the clusters, which are not justified by the applied setting being considered.

We are not the first to recognize this problem, and there is literature attempting to address issues with kernel robustness in model-based clustering. One direction is to choose a flexible class of kernels, which can characterize a wide variety of densities. For example, one can replace the Gaussian kernel with one that accommodates asymmetry, skewness and/or heavier tails (Karlis and Santourian (2009); Juárez and Steel (2010); O’Hagan et al. (2016); Gallaugher and McNicholas (2018); among others). A related direction is to nonparametrically estimate the kernels specific to each cluster, while placing minimal constraints for identifiability, such as unimodality and sufficiently light tails. This direction is related to the mode-based clustering algorithms of Li et al. (2007); see also Rodríguez and Walker (2014) for a Bayesian approach using unimodal kernels. Unfortunately, as discussed by Hennig et al. (2015), a kernel that is too flexible leads to ambiguity in defining a cluster and identifiability issues: for example, one cluster can be the union of several clusters that are close. Practically, such flexible kernels demand a large number of parameters, leading to daunting computation cost.

A promising new strategy is to replace the likelihood with a robust alternative. Coretto and Hennig (2016) propose a pseudo-likelihood based approach for robust multivariate clustering, which captures outliers with an extra improper uniform component. Miller and Dunson (2018) propose a coarsened Bayes approach for robustifying Bayesian inference and apply it to clustering problems. Instead of assuming that the observed data are exactly generated from (1) in defining a Bayesian approach, they condition on the event that the empirical probability mass function of the observed data is within some small neighborhood of that for the assumed model. Both of these methods aim to allow small deviations from a simple kernel. It is difficult to extend these approaches to data with high complexity, such as clustering multiple time series, images, etc.

We propose a new approach based on a Bayesian model for the pairwise distances, avoiding a complete specification of the likelihood function for the data *y*_(*n*)_. There is a rich literature proposing Bayesian approaches that replace an exact likelihood function with some alternative. Chernozhukov and Hong (2003) consider a broad class of such quasi-posterior distributions. Jeffreys (1961) proposed a substitution likelihood for quantiles for use in Bayesian inference; also refer to Dunson and Taylor (2005). Hoff (2007) proposed a Bayesian approach to inference in copula models, which avoids specifying models for the marginal distributions via an extended rank likelihood. Johnson (2005) proposed Bayesian tests based on modeling frequentist test statistics instead of the data directly. These are just some of many examples.

Our proposed Bayesian distance clustering approach gains some of the advantages of model-based clustering, such as uncertainty quantification and flexibility, while significantly simplifying the model specification task. There is a connection between our approach and nonnegative matrix factorization (NMF) methods (Kuang et al., 2012; Zhao et al., 2015; Kuang et al., 2015). Certain NMF algorithms can be viewed as fast approximations to our likelihood-based approach. Our major contributions are: (i) establishing a novel link between model- and distance-based frameworks, (ii) introducing a principled choice for assigning kernels for distances (equivalent to the affinity/similarity score in NMFs), and (iii) providing a way to calibrate the parameters within the proposed probabilistic framework.

## 2. Partial likelihood for distances

### 2.1 Motivation for partial likelihood

Suppose that data *y*_(*n*)_ are generated from model (1) or equivalently (2). We focus on the case in which $y_{i}={\left(y_{i,1},\ldots ,y_{i,p}\right)}^{\prime }\in Y\subset {\mathbb{R}}^{p}$. The conditional likelihood of the data *y*_(*n*)_ given clustering indices *c*_(*n*)_ can be expressed as

(3) $$L\left(y_{\left(n\right)};c_{\left(n\right)}\right)=\prod_{h=1}^{k}\prod_{i:c_{i}=h}K_{h}\left(y_{i}\right)=\prod_{h=1}^{k}L_{h}\left(y^{\left[h\right]}\right),$$

where we let $K_{h}(y)$ denote the density of data within cluster *h*, and $y^{\left[h\right]}=\left\{y_{i}:c_{i}=h\right\}=\left\{y_{i}^{\left[h\right]},i=1,\ldots ,n_{h}\right\}$ is the data in cluster *h*. Since the information of *c*_(*n*)_ is stored by the index with [*h*], we will omit *c*_(*n*)_ in the notation when [*h*] appears. Referring to $y_{1}^{\left[h\right]}$ as the *seed* for cluster *h*, we can express the likelihood *L*_*h*_(*y*^[*h*]^) using the change-of-variables $\left(y_{1}^{\left[h\right]},y_{2}^{\left[h\right]}\ldots ,y_{n_{h}}^{\left[h\right]}\right)\to \left(y_{1}^{\left[h\right]},{\tilde{d}}_{2,1}^{\left[h\right]},\ldots ,{\tilde{d}}_{n_{h},1}^{\left[h\right]}\right)$:

(4) $$K_{h}\left(y_{1}^{\left[h\right]}\right)\prod_{i=2}^{n_{h}}G_{h}\left({\tilde{d}}_{i,1}^{\left[h\right]}|y_{1}^{\left[h\right]}\right)\;=K_{h}\left(y_{1}^{\left[h\right]}|{\tilde{d}}_{2,1}^{\left[h\right]},\ldots ,{\tilde{d}}_{n_{h},1}^{\left[h\right]}\right)\;G_{h}\left({\tilde{d}}_{2,1}^{\left[h\right]},\ldots ,{\tilde{d}}_{n_{h},1}^{\left[h\right]}\right),$$

where ${\tilde{d}}_{i,1}^{\left[h\right]}=y_{i}^{\left[h\right]}-y_{1}^{\left[h\right]}$ denotes the difference vector between $y_{i}^{\left[h\right]}$ and $y_{1}^{\left[h\right]}$ (with *G* the transformed kernel), and the second line is an equivalent factorization of the joint distribution, with $K_{h}\left(y_{1}^{\left[h\right]}|.\right)$ the conditional density of $y_{1}^{\left[h\right]}$ given the differences. Expression (4) is a product of the densities of the seed and (*n*_*h*_ − 1) differences. As the cluster size *n*_*h*_ increases, the relative contribution of the seed density $K_{h}\left(y_{1}^{\left[h\right]}|.\right)$ will decrease and the likelihood becomes dominated by *G*_*h*_. With this heuristic justification, we discard the $K_{h}\left(y_{1}^{\left[h\right]}|.\right)$ term by treating the value of $y_{1}^{\left[h\right]}$ as random and integrating out $K_{h}\left(y_{1}^{\left[h\right]}|.\right)$.

We now use a toy example to illustrate how to *derive* the function *G*_*h*_ from a known model-based likelihood (later we will show how to *specify G*_*h*_ directly, when the model-based likelihood is not known). Consider the case of $y_{i}\in \mathbb{R}$ from a Gaussian mixture, starting from the likelihood of those *y*_*i*_’s associated with *c*_*i*_ = *h*:

$$L_{h}\left(y^{\left[h\right]}\right)={\left(2\pi \sigma_{h}^{2}\right)}^{-n_{h}/2}\text{exp}\left[-\frac{\sum_{i=1}^{n_{h}}{\left(y_{i}^{\left[h\right]}-\mu_{h}\right)}^{2}}{2\sigma_{h}^{2}}\right]\;={\left(2\pi \sigma_{h}^{2}\right)}^{-n_{h}/2}\text{exp}\left\{-\frac{\sum_{i=1}^{n_{h}}\left[{\left(y_{i}^{\left[h\right]}-y_{1}^{\left[h\right]}\right)}^{2}+{\left(y_{1}^{\left[h\right]}-\mu_{h}\right)}^{2}+2\left(y_{i}^{\left[h\right]}-y_{1}^{\left[h\right]}\right)\left(y_{1}^{\left[h\right]}-\mu_{h}\right)\right]}{2\sigma_{h}^{2}}\right\},$$

To obtain *G*_*h*_, based on the formula f(d,y)=f(y∣d)∫f(d,y)dy, we use the change-of-variable ${\tilde{d}}_{i,1}^{\left[h\right]}=y_{i}^{\left[h\right]}-y_{1}^{\left[h\right]}$, and integrate out $y_{1}^{\left[h\right]}$ [as the information of $y_{1}^{\left[h\right]}$ is now in $K_{h}\left(y_{1}^{\left[h\right]}|{\tilde{d}}_{2,1}^{\left[h\right]},\ldots ,{\tilde{d}}_{n_{h},1}^{\left[h\right]}\right)$]:

$$G_{h}\left({\tilde{d}}_{2,1}^{\left[h\right]},\ldots ,{\tilde{d}}_{n_{h},1}^{\left[h\right]}\right)=\;\int {\left(2\pi \sigma_{h}^{2}\right)}^{-n_{h}/2}\text{exp}\left[-\frac{\sum_{i=1}^{n_{h}}{\tilde{d}}_{i,1}^{[h]2}+n_{h}{\left(y_{1}^{\left[h\right]}-\mu_{h}\right)}^{2}+2\left(y_{1}^{\left[h\right]}-\mu_{h}\right)\sum_{i=1}^{n_{h}}{\tilde{d}}_{i,1}^{\left[h\right]}}{2\sigma_{h}^{2}}\right]\text{d}y_{1}^{\left[h\right]}\;={\left(2\pi \sigma_{h}^{2}\right)}^{-\left(n_{h}-1\right)/2}\frac{1}{\sqrt{n_{h}}}\text{exp}\left[-\frac{\sum_{i=1}^{n_{h}}{\tilde{d}}_{i,1}^{[h]2}-{\left(\sum d_{i,1}^{\left[h\right]}\right)}^{2}/n_{h}}{2\sigma_{h}^{2}}\right]\;\overset{\left(a\right)}{=}{\left(2\pi \sigma_{h}^{2}\right)}^{-\left(n_{h}-1\right)/2}\frac{1}{\sqrt{n_{h}}}\prod_{i,j}\text{exp}\left(-\frac{{\tilde{d}}_{i,j}^{[h]2}}{4n_{h}\sigma_{h}^{2}}\right),$$

where (*a*) is due to $2\sum_{i<j}{\tilde{d}}_{\text{ij}}^{[h]2}=\sum_{i}\sum_{j}{\left({\tilde{d}}_{i,1}^{\left[h\right]}-{\tilde{d}}_{j,1}^{\left[h\right]}\right)}^{2}=n_{h}\sum_{i}{\tilde{d}}_{i,1}^{[h]2}+n_{h}\sum_{j}{\tilde{d}}_{j,1}^{[h]2}-\sum_{j\neq i}2{\tilde{d}}_{i,1}^{\left[h\right]}{\tilde{d}}_{j,1}^{\left[h\right]}-\sum_{i=j}2{\tilde{d}}_{i,1}^{\left[h\right]}{\tilde{d}}_{j,1}^{\left[h\right]}=2n_{h}\left[\sum {\tilde{d}}_{i,1}^{[h]2}-{\left(\sum {\tilde{d}}_{i,1}^{\left[h\right]}\right)}^{2}/n_{h}\right]$.

In the above example, note that the right hand side appears to be a product density of *all* the pairwise differences ${\tilde{D}}^{\left[h\right]}={\left\{{\tilde{d}}_{i,j}^{\left[h\right]}\right\}}_{\left(i,j\right)}$, besides those formed with the seed. This is due to the linear equality that ${\tilde{d}}_{i,j}^{\left[h\right]}={\tilde{d}}_{i,1}^{\left[h\right]}-{\tilde{d}}_{j,1}^{\left[h\right]}$ — therefore, there are effectively only (*n*_*h*_ − 1) free random variables; once they are given, the rest are completely determined.

Generalizing from this form, we now specify *G* as

(5) $$G_{h}\left({\tilde{d}}_{2,1}^{\left[h\right]},\ldots ,{\tilde{d}}_{n_{h},1}^{\left[h\right]}\right)=\prod_{i=1}^{n_{h}}\prod_{j>i}g_{h}^{1/n_{h}}\left({\tilde{d}}_{i,j}^{\left[h\right]}\right),$$

where $g_{h}:{\mathbb{R}}^{p}\to {\mathbb{R}}_{+}$ and each ${\tilde{d}}_{i,j}^{\left[h\right]}$ is assigned a marginal density. The power 1/*n*_*h*_ is a calibration parameter that adjusts the order discrepancy between the numbers of (*n*_*h*_ − 1)*n*_*h*_/2 marginal densities and (*n*_*h*_ − 1) effective random variables. We will formally justify this calibration in the theory section.

**Remark 1** *To clarify*, *despite the simple product form*, (5) *should not be treated as the independent densities of n*_*h*_(*n*_*h*_ −1)/2 *differences. This is because these differences contain effectively* (*n*_*h*_ −1) *random variables* ${\tilde{d}}_{2,1}^{\left[h\right]},\ldots ,{\tilde{d}}_{n_{h},1}^{\left[h\right]}$, *and* (*n*_*h*_ − 1)(*n*_*h*_ − 2)/2 *interaction terms* ${\tilde{d}}_{i,j}^{\left[h\right]}={\tilde{d}}_{i,1}^{\left[h\right]}-{\tilde{d}}_{j,1}^{\left[h\right]}$; *these interaction terms induce dependence between* ${\tilde{d}}_{i,1}^{\left[h\right]}$ *and* ${\tilde{d}}_{j,1}^{\left[h\right]}$:

$$G_{h}\left({\tilde{d}}_{2,1}^{\left[h\right]},\ldots ,{\tilde{d}}_{n_{h},1}^{\left[h\right]}\right)=\left[\prod_{i=2}^{n_{h}}g_{h}^{1/n_{h}}\left({\tilde{d}}_{i,1}^{\left[h\right]}\right)\right]\left[\prod_{i=1}^{n_{h}-1}\prod_{i<j}g_{h}^{1/n_{h}}\left({\tilde{d}}_{i,1}^{\left[h\right]}-{\tilde{d}}_{j,1}^{\left[h\right]}\right)\right].$$

*For example*, *for* ${\tilde{d}}_{2,1}^{\left[h\right]}$ *and* ${\tilde{d}}_{3,1}^{\left[h\right]}$, *the related terms in* (5) *are:*

$$g^{1/n_{h}}\left({\tilde{d}}_{2,1}^{\left[h\right]}\right)g^{1/n_{h}}\left({\tilde{d}}_{3,1}^{\left[h\right]}\right)g^{1/n_{h}}\left({\tilde{d}}_{2,1}^{\left[h\right]}-{\tilde{d}}_{3,1}^{\left[h\right]}\right),$$

*which is a non*-*separable function of* ${\tilde{d}}_{2,1}^{\left[h\right]}$ *and* ${\tilde{d}}_{3,1}^{\left[h\right]}$, *and hence not independent*.

We now state the assumptions that we use for clustering.

**Assumption 1** *For those data within a cluster*, $y_{i}^{\left[h\right]}$ *and* $\;y_{j}^{\left[h\right]}$ *are independent and identically distributed*.

Focusing on marginally specifying each *g*_*h*_, we can immediately obtain two key properties of ${\tilde{d}}_{i,j}^{\left[h\right]}=y_{i}^{\left[h\right]}-y_{j}^{\left[h\right]}$: (1) Expectation zero, and (2) Marginal symmetry with skewness zero. Hence, the distribution of the differences is substantially simpler than the original data distribution $K_{h}$. This suggests using *G*_*h*_ for clustering will substantially reduce the model complexity and improve robustness.

We connect the density of the differences to a likelihood of ‘distances’ — here used as a loose notion including metrics, semi-metrics and divergences. Consider *d*_*i,j*_ ∈ [0, ∞) as a transform of ${\tilde{d}}_{i,j}$, such as some norm $d_{i,j}=\left\|{\tilde{d}}_{i,j}\right\|$ (e.g. Euclidean or 1-norm); hence, a likelihood for *d*_*i,j*_ is implicitly associated to a pushforward measure from the one on ${\tilde{d}}_{i,j}$ (assuming a measurable transform). For example, an exponential density on $d_{i,j}={\left\|{\tilde{d}}_{i,j}\right\|}_{1}$ can be taken as the result of assigning a multivariate Laplace on ${\tilde{d}}_{i,j}$. We can further generalize the notion of difference from subtraction to other types, such as ratio, cross-entropy, or an application-driven specification (Izakian et al., 2015).

To summarize, this motivates the practice of first calculating a matrix of pairwise distances, and then assigning a partial likelihood for clustering. For generality, we slightly abuse notation and replace the difference array $\tilde{D}$ with the distance matrix *D* in (5), and denote the density by *G*_*h*_(*D*^[*h*]^). We will refer to (5) as the *distance likelihood* from now on. Conditional on the clustering labels,

(6) $$L\left[D;c_{\left(n\right)}\right]=\prod_{h=1}^{k}G_{h}\left(D^{\left[h\right]}\right),$$

with $c_{i}\sim \sum_{h=1}^{k}\pi_{h}\delta_{h}$ independently, as is (2).

**Figure 1:**
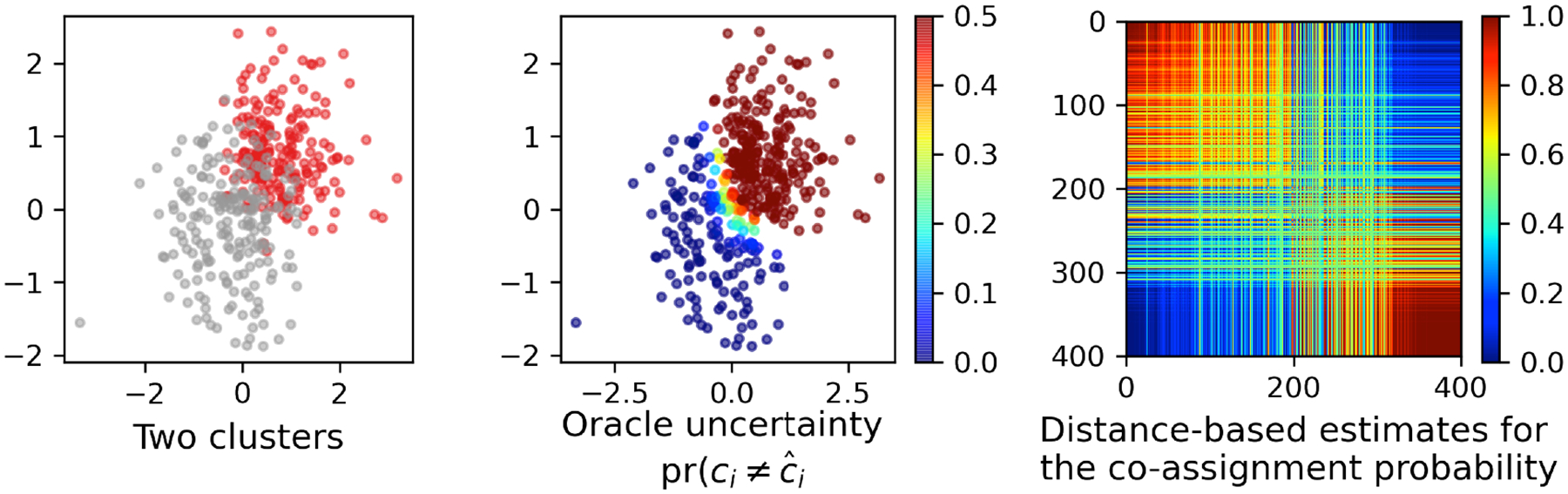
Illustration of the clustering uncertainty and its estimation using the distance-based clustering method. Left panel: two overlapping clusters (red and grey) generated from two skew Gaussian distributions with *n* = 400. Center panel: the oracle uncertainty $\text{pr}\left(c_{i}\neq {\hat{c}}_{i}|y_{i}\right)$ calculated based on the generative distribution. Right panel: the matrix of the co-assignment probabilities pr(*c*_*i*_ = *c*_*j*_ | *D*) estimated using the distance likelihood of *D*.

To provide some intuition about the clustering uncertainty, we simulate two clusters using the bivariate skewed Gaussian distribution: in each dimension, the first cluster has a skewness of 4 (red in Figure 1, left panel), location of 0 and scale of 1, and the second has a skewness of −2, location of 0.5 and scale of 1 (grey in Figure 1, left panel); the two sub-coordinates are generated independently. Via the skew Gaussian density $K_{h}(y_{i})$ used to generate the data, we can compute the oracle assignment probability pr(*c*_*i*_ = *h* | *y*_*i*_) for *h* = 1 and 2, and the most likely cluster assignment ${\hat{c}}_{i}$ for each data point.

Clearly, due to the overlapping of the two clusters, there is a significant amount of uncertainty for those near the location (0, 0), as the $\text{pr}\left(c_{i}\neq {\hat{c}}_{i}\right)$ remains away from 0 (Figure 1, center panel) — importantly, such uncertainty does not vanish even as *n* → ∞, as these points will remain nearly equidistant to the two cluster centers. Using the distance likelihood on *D*, we can obtain an easy quantification of the uncertainty, by sampling *c*_*i*_ from the posterior distribution and calculating the co-assignment probabilities pr(*c*_*i*_ = *c*_*j*_ | *D*); as shown in the right panel, the off-diagonal block shows that a significant portion of data that can be co-assigned to either the first or the second cluster with a non-trivial probability.

### 2.2 Choosing a distance density for clustering

To implement our Bayesian distance clustering approach, we need a definition of clusters, guiding us to choose a parametric form for *g*_*h*_(.) in (5). For conciseness, we will focus on the norm-based distances from now on. A popular intuition for a cluster is a group of data points, such that most of the distances among them are relatively small. That is, the probability of finding large distances within a cluster should be low. We now state the assumption.

**Assumption 2** *With σ*_*h*_ > 0, *a scale parameter and ϵ*_*h*_ *a function that rapidly declines towards* 0 *as t increases*.

(7) $$\text{pr}\left(d_{i,j}^{\left[h\right]}\geq t\sigma_{h}\right)\leq \epsilon_{h}(t)\text{ for sufficiently large }t>0.$$

For such a decline, it is common to consider the sub-exponential rate (Wainwright, 2019), $\epsilon_{h}(t)=O\{\text{exp}(-t/b)\}$ with some constant *b* > 0. Ideally, we want *σ*_*h*_ to be small, so that most of the distances within a cluster are small.

It is tempting to consider using a simple exponential density Exp(*σ*_*h*_) for modeling $d_{i,j}^{\left[h\right]}$, however, we make an important observation here: the exponential distribution has a deterministic relationship between the mean *σ*_*h*_ and the variance $\sigma_{h}^{2}$ — this means any slightly large $Ed_{i,j}^{\left[h\right]}$ (such as when the distribution of $d_{i,j}^{\left[h\right]}$ does not follow a exponential decay near zero) will inflate the estimate of *σ*_*h*_, making it difficult to use small distances for clustering.

This motivates us to use a two-parameter distribution instead — in this article, we use Gamma (*α*_*h*_, *σ*_*h*_) for *g*_*h*_ in (5), whose variance $\alpha_{h}\sigma_{h}^{2}$ is no longer completely determined by the mean *α*_*h*_*σ*_*h*_.

(8) $$g_{h}\left(d_{i,j}^{\left[h\right]}\right)=\frac{1}{\Gamma \left(\alpha_{h}\right)\sigma_{h}^{\alpha_{h}}}x^{\alpha_{h}-1}\text{ exp }\left(-d_{i,j}^{\left[h\right]}/\sigma_{h}\right).$$

We defer the prior choice for *α*_*h*_ and *σ*_*h*_ to a later section. For now, we verify that the Gamma distribution does have a sub-exponential tail that satisfies Assumption 1.

**Lemma 2** *(Bound on the right tail) If d has the density* (8), *for any α*_*h*_ *≥* 1 *and t* > 0,

$$\text{pr}\left(d\geq t\sigma_{h}\right)\leq Mt^{\alpha_{h}}\text{ exp }(-t),$$

*where* $M={\left(\alpha_{h}\right)}^{-\alpha_{h}}\text{exp}\left(\alpha_{h}\right)$.

**Remark 3** *The polynomial term $t^{\alpha_{h}}$ allows deviation from the exponential distribution at small t; its effect vanishes as t increases*.

The assumption on the distances are connected to some implicit assumptions on the data distribution $K\left(y_{i}\right)$. As such a link varies with the specific form of the distance, we again focus on the vector norm of subtraction $d_{i,j}^{\left[h\right]}={\left\|y_{i}^{\left[h\right]}-y_{j}^{\left[h\right]}\right\|}_{q}$, with $\|x{\|}_{q}={\left(\sum_{j=1}^{p}x_{j}^{q}\right)}^{1/q}$ and *q* ≥ 1. We now show that a sub-exponential tail for the vector norm distance is a necessary result of assuming sub-exponential tails in $K\left(y_{i}\right)$.

**Lemma 4** *(Tail of vector norm distance) If there exist bound constants $m_{1}^{\left[h\right]}$*, $m_{2}^{\left[h\right]}>0$, *such that for all j* = 1, …, *p*

(9) $$\text{pr}\left(\left|y_{i,j}^{\left[h\right]}-Ey_{i,j}^{\left[h\right]}\right|\geq t\right)\leq m_{1}^{\left[h\right]}\text{ exp}\left(-m_{2}^{\left[h\right]}t\right),$$

*then*, *there exist another two constants ν*_*h*_, *b*_*h*_ > 0, *such that for any q ≥* 1

(10) $$\text{pr}\left(d_{\text{ij}}^{\left[h\right]}>tb_{h}p^{\eta }\right)\leq 2p\text{ exp}\left\{-tp^{\left(\eta -1/q\right)}/2\right\}\;\text{for }t>2p^{1/q-\eta }\nu_{h}^{2}.$$

**Remark 5** *The concentration property* (9) *is less restrictive than common assumptions on the kernel in a mixture model*, *such as Gaussianity*, *log*-*concavity or unimodality*.

## 3. Hyper-prior specification for *α*_*h*_ and *σ*_*h*_

In Bayesian clustering, it is useful to choose the prior parameters in a reasonable range (Malsiner-Walli et al., 2017). Recall in our gamma density, *α*_*h*_ determines the mean for $d_{i,j}^{\left[h\right]}$ at *α*_*h*_*σ*_*h*_. To favor small values for the mode while accommodating a moderate degree of uncertainty, we use a Gamma prior *α*_*h*_ ~ Gamma(1.5, 1.0).

To select a prior for *σ*_*h*_, we associate it with a pre-specified maximum cluster number *k*. We can view *k* as a packing number — that is, how many balls (clusters) we can fit in a container of the data. To formalize, imagine a *p*-dimensional ellipsoid in ${\mathbb{R}}^{p}$ enclosing all the observed data. The smallest volume of such an ellipsoid is

$$\text{vol}\left(\text{Data}\right)=M\;\underset{\;\mu \in {\mathbb{R}}^{p},Q≻0}{\text{min}}{\left(\text{det }Q\right)}^{-1/2},\text{ s.t. }{\left(y_{i}-\mu \right)}^{\text{T}}Q\left(y_{i}-\mu \right)\leq 1\text{ for }i=1,\ldots ,n,$$

which can be obtained via a fast convex optimization algorithm (Sun and Freund, 2004), with $M={\tilde{\pi }}^{p/2}/\Gamma (p/2+1)$ and $\tilde{\pi }\approx 3.14$.

If we view each cluster as a high-probability ball of points originating from a common distribution, then the diameter — the distance between the two points that are farthest apart — is ~ 4*σ*_*h*_. This is calculated based on pr(*d* ≤ 4*σ*_*h*_) *≈* 0.95 using the gamma density with shape 1.5 (the prior mean of *α*_*h*_). We denote the ball by $B_{2\sigma_{h}}$, with $\text{vol}\left(B_{2\sigma_{h}}\right)=M{\left(2\sigma_{h}\right)}^{p}$.

Setting *k* to the packing number

$$k≃\frac{\text{vol}\left(\text{Data}\right)}{\text{vol}\left(B_{2\sigma_{h}}\right)}$$

yields a sensible prior mean for *σ*_*h*_. For conjugacy, we choose an inverse-gamma prior for *σ*_*h*_ with $E\left(\sigma_{h}\right)=\beta_{h}$,

$$\sigma_{h}\sim \text{Inverse-Gamma}\left(2,\beta_{\sigma }\right),\;\beta_{\sigma }=\frac{1}{2}{\left\{\frac{\text{vol}\left(\text{Data}\right)}{\text{kM}}\right\}}^{1/p}.$$

The above prior can be used as a default in broad applications, and does not require tuning to each new application.

## 4. Theory

We describe several interesting properties for the distance likelihood.

### 4.1 Calibration

**Lemma 6** (Exchangeability) *When the product density* (5) *is used for all G*_*h*_(*D*^[*h*]^), *h* = 1, …, *k*, *the distance likelihood* (6) *is invariant to permutations of the indices i:*

$$L\left\{y_{\left(n\right)};c_{\left(n\right)}\right\}=L\left\{y_{\left(n^{*}\right)};c_{\left(n^{*}\right)}\right\},$$

*with* (n*) = {1_*_, …, n_*_} *denoting a set of permuted indices*.

We fill a missing gap between the model-based and distance likelihoods by considering an information-theoretic analysis of the two clustering approaches. This also leads to a principled choice of the power 1/*n*_*h*_ in (5).

To quantify the information in clustering, we first briefly review the concept of Bregman divergence (Bregman, 1967). Letting ϕ:S→ℝ be a strictly convex and differentiable function, with S the domain of *ϕ*, the Bregman divergence is defined as

$$B_{\phi }(x,y)=\phi (x)-\phi (y)-{\left(x-y\right)}^{\text{T}}\nabla \phi (y),$$

where ▽*ϕ*(*y*) denotes the gradient of *ϕ* at *y*. A large family of loss functions, such as squared norm and Kullback-Leibler divergence, are special cases of the Bregman divergence with suitable *ϕ*. For model-based clustering, when the regular exponential family (‘regular’ as the parameter space is a non-empty open set) is used for the component kernel $K_{h}$, Banerjee et al. (2005) show that there always exists a re-parameterization of the kernel using Bregman divergence. Using our notation,

$$K_{h}\left(y_{i};\theta_{h}\right)=\text{exp}\left\{T{\left(y_{i}\right)}^{\prime }\theta_{h}-\psi \left(\theta_{h}\right)\right\}\kappa \left(y_{i}\right)\Leftrightarrow \text{exp}\left[-B_{\phi }\left\{T\left(y_{i}\right),\mu_{h}\right\}\right]b_{\phi }\left\{T\left(y_{i}\right)\right\},$$

where *T* (*y*_*i*_) is a transformation of *y*_*i*_, in the same form as the minimum sufficient statistic for *θ*_*h*_ (except this ‘statistic’ is based on only one data point *y*_*i*_); *μ*_*h*_ is the expectation of *T*(*y*_*i*_) taken with respect to $K_{h}\left(y;\theta_{h}\right)$; *ψ*, *κ* and *b*_*ϕ*_ are functions mapping to (0, ∞).

With this re-parameterization, maximizing the model-based likelihood over *c*_(*n*)_ becomes equivalent to minimizing the within-cluster Bregman divergence

$$H_{y}=\sum_{h=1}^{k}H_{y}^{\left[h\right]},\;H_{y}^{\left[h\right]}=\sum_{i=1}^{n_{h}}B_{\phi }\left\{T\left(y_{i}^{\left[h\right]}\right),\mu_{h}\right\}.$$

We will refer to *H*_*y*_ as the model-based divergence.

For the distance likelihood, considering those distances that can be viewed or re-parameterized as a pairwise Bregman divergence, we assume each $g\left(d_{i,j}^{\left[h\right]}\right)$ in the distance likelihood (5) can be re-written with a calibrating power *β*_*h*_ > 0 as

$$g^{\beta_{h}}\left(d_{i,j}^{\left[h\right]}\right)=z^{\beta_{h}}\text{ exp }\left[-\beta_{h}B_{\phi }\left\{T\left(y_{i}^{\left[h\right]}\right),T\left(y_{j}^{\left[h\right]}\right)\right\}\right],$$

with *z* > 0 the normalizing constant. A distance-based divergence *H*_*d*_ can be computed as

(11) $$H_{d}=\sum_{h=1}^{k}H_{d}^{\left[h\right]},\;H_{d}^{\left[h\right]}=\beta_{h}\sum_{i=1}^{n_{h}}\sum_{j=1}^{n_{h}}\frac{1}{2}B_{\phi }\left\{T\left(y_{i}^{\left[h\right]}\right),T\left(y_{j}^{\left[h\right]}\right)\right\}.$$

We now compare these two divergences *H*_*y*_ and *H*_*d*_ at their expectations.

**Lemma 7** *(Expected Bregman Divergence) The distance*-*based Bregman divergence* (11) *in cluster h has*

$$E_{y^{\left[h\right]}}H_{d}^{\left[h\right]}=\beta_{h}E_{y_{i}^{\left[h\right]}}E_{y_{j}^{\left[h\right]}}\sum_{i=1}^{n_{h}}\sum_{j=1}^{n_{h}}\frac{1}{2}B_{\phi }\left\{T\left(y_{i}^{\left[h\right]}\right),T\left(y_{j}^{\left[h\right]}\right)\right\}\;=\left(n_{h}\beta_{h}\right)E_{y^{\left[h\right]}}\;[\sum_{i=1}^{n_{h}}\frac{B_{\phi }\left\{T\left(y_{i}^{\left[h\right]}\right),\mu_{h}\right\}+B_{\phi }\left\{\mu_{h},T\left(y_{i}^{\left[h\right]}\right)\right\}}{2}],$$

*where the expectation over y*^[*h*]^ *is taken with respect to $K_{h}$*.

**Remark 8** *The term inside the expectation on the right hand side is the symmetrized Bregman divergence between $T\left(y_{i}^{\left[h\right]}\right)$ and μ*_*h*_ (Banerjee et al., 2005)*. Therefore*, $E_{y^{\left[h\right]}}H_{d}^{\left[h\right]}=\left(n_{h}\beta_{h}\right)E_{y^{\left[h\right]}}H_{y}^{\left[h\right]}$ *when B*_*ϕ*_(·, ·) *is symmetric*.

There is an order difference of $O\left(n_{h}\right)$ between distance-based and model-based divergences. Therefore, a sensible choice is simply setting *β*_*h*_ = 1/*n*_*h*_. This power is related to the weights used in composite pairwise likelihood (Lindsay, 1988; Cox and Reid, 2004).

### 4.2 Relationship to Graph Cut

It is also interesting to consider the matrix form of the distance likelihood. We use *C* as an *n* × *k* binary matrix encoding the cluster assignment, with *C*_*i,h*_ = 1 if *c*_*i*_ = *h*, and all other *C*_*i,h′*_ = 0. Then it can be verified that *C*^T^*C* = diag(*n*_1_, …, *n*_*k*_). Hence the distance likelihood, with the Gamma density, is

(12) $$G(D;C)\propto \text{exp}\left[\text{tr}\left\{C^{\text{T}}(\text{log }D)C\Lambda {\left(C^{\text{T}}C\right)}^{-1}\right\}\right]\text{ exp }\left[-\text{tr}\left\{C^{\text{T}}\text{DC}{\left(\Sigma C^{\text{T}}C\right)}^{-1}\right\}\right],$$

where *D* is the *n* × *n* distance matrix, log is applied element-wise, Σ diag(*σ*_1_, …, *σ*_*k*_), and Λ = diag(*α*_1_ *−* 1, …, *α*_*h*_ *−* 1). If *C* contains zero columns, the inverse is replaced by a generalized inverse.

One may notice some resemblance of (12) to the loss function in graph partitioning algorithms. Indeed, if we simplify the parameters to *α*_1_ = ··· = *α*_*k*_ = *α*_0_ and *σ*_1_ = ··· = *σ*_*k*_ = *σ*_0_, then

(13) $$G(D;C)\propto \text{exp }\left[\text{tr}\left\{C^{\text{T}}\text{AC}{\left(C^{\text{T}}C\right)}^{-1}\right\}\right],$$

where *A* = *κ***1**_*n,n*_ − *D*/*σ*_0_ + (*α*_0_ − 1) log *D* can be considered as an adjacency matrix of a graph formed by a log-Gamma distance kernel, with **1**_*n,n*_ as an *n*×*n* matrix with all elements equal to 1; *κ* a constant so that each *A*_*i,j*_ > 0 (since *κ* enters the likelihood as a constant tr{*C*^T^*κ***1**_*n,n*_*C*(*C*^T^*C*)^−1^} = *nκ*, it does not impact the likelihood of *C*). To compare, the popular normalized graph-cut loss (Bandeira et al., 2013) is

(14) $$\text{NCut-Loss }=\sum_{h=1}^{k}\sum_{i:c_{i}=h}\sum_{j:c_{j}\neq h}\frac{A_{i,j}}{2n_{h}},$$

which is the total edges deleted because of partitioning (weighted by $n_{h}^{-1}$ to prevent trivial cuts). There is an interesting link between (13) and (14).

**Lemma 9** *Considering a graph with weighted adjacency matrix A*, *the normalized graph*-*cut loss is related to the negative log*-*likelihood (omitting constant)* (13) *via*

$$2\text{NCut-Loss }=-\text{tr}\left\{C^{\text{T}}\text{AC}{\left(C^{\text{T}}C\right)}^{-1}\right\}+\overset{n}{\underset{i=1}{\sum }}\frac{\sum_{j=1}^{n}A_{i,j}}{n_{c_{i}}}.$$

**Remark 10** *The difference on the right is often known as the degree*-*based regularization (with $\sum_{j=1}^{n}A_{i,j}$ the degree*, $n_{c_{i}}$ *the size of the cluster that data i is assigned to). When the cluster sizes are relatively balanced*, *we can ignore its effect*.

Such a near-equivalence suggests that we can exploit popular graph clustering algorithms, such as spectral clustering, for good initiation of *C* as a warm-start of the Markov chain Monte Carlo algorithm.

## 5. Posterior computation

For the posterior computation, Gibbs sampler is easy to derive as it involves updating one element of the parameter at a time, from the full conditional distribution. However, this could lead to a heavy computational cost for our model. To understand this, consider the update step for each *c*_*i*_, which involves a draw from the categorical distribution:

$$\text{pr}\left(c_{i}=h|.\right)=\frac{\pi_{h}G\left(D;{\tilde{C}}_{i,h}\right)}{\sum_{h^{\prime }=1}^{k}\pi_{h^{\prime }}G\left(D;{\tilde{C}}_{i,h^{\prime }}\right)},$$

where ${\tilde{C}}_{h}$ denotes a matrix equal to the current value of *C*, except replacing the *i*th row with *C*_*i,h*_ = 1 and *C*_*i,j*_ = 0 for other *j* ≠ *h*. Since *G*(*D*; *C*) involves a matrix inverse term (*C*^T^*C*)^−1^, the above ratio cannot be simplified to reduce the computational burden. The evaluation cost for each *G*(*D*; *C*) is dominated by the matrix multiplication steps within, hence having an overall cost of $O\left(n^{2}k\right)$. Therefore, iterating over *h* = 1, …, *k* and *i* = 1, …, *n* will lead to a high cost in one sweep of update.

To solve this problem, we instead develop a more efficient algorithm based on the approximate Hamiltonian Monte Carlo (HMC) algorithm. We use a continuous relaxation of each row *C*_*i*_ (on a simplex vertex) into the interior of the simplex, and denote the relaxation by $W_{i}\in \Delta_{\backslash \partial }^{\left(k-1\right)}$. This is achieved via a tempered softmax re-parameterization (Maddison et al., 2017)

$$w_{i,h}=\frac{\text{exp}\left(v_{i,h}/t\right)}{\sum_{h^{\prime }=1}^{k}\text{exp}\left(v_{i,h^{\prime }}/t\right)},\;h=1,\ldots ,k.$$

At small *t* > 0 and close to 0, if one *v*_*i,h*_ is slightly larger than the rest in {*v*_*i,*1_, …, *v*_*i,k*_}, then *w*_*i,h*_ will be close to 1, and all the other *w*_*i,h*__′_ ’s close to 0. In this article, we use *t* = 0.1 as a balance between the approximation accuracy and the numeric stability of the algorithm. In addition, we re-parameterize the other parameters using the softplus function $\sigma_{h}=\text{log}\left[\text{exp}\left({\tilde{\sigma }}_{h}\right)+1\right]$, $\alpha_{h}=\text{log}\left[\text{exp}\left({\tilde{\alpha }}_{h}\right)+1\right]$ for *h* = 1, …, *k*, and and the softmax function $\left(\pi_{1},\ldots ,\pi_{k}\right)=\text{softmax}\left({\tilde{\pi }}_{1},\ldots ,{\tilde{\pi }}_{k}\right)$ (as defined above except with *t* = 1), where ${\tilde{\sigma }}_{h}$, ${\tilde{\alpha }}_{h}$ and ${\tilde{\pi }}_{h}$ are all unconstrained parameters in ℝ amenable to the off-the-shelf continuous HMC algorithm.

We denote the vectorized parameters by $\beta =\left(\upsilon_{1,1},\ldots ,\upsilon_{n,k},{\tilde{\sigma }}_{1},\ldots ,{\tilde{\sigma }}_{k},{\tilde{\alpha }}_{1},\ldots ,{\tilde{\alpha }}_{k},{\tilde{\pi }}_{1},\ldots ,{\tilde{\pi }}_{k}\right)$. To sample from posterior distribution *β* ~ Π_*β*|*D*_(*·*), the HMC uses an auxiliary momentum variable *v* and samples from a joint distribution Π(*β*, *v*) = Π(*β* | *D*)Π(*v*), where a common choice of Π(*v*) is the density of N(0, *M*). Denote *U*(*β*) = −log Π(*β* | *D*) and *K*(*v*) = −log *π*(*v*) = *v*^*T*^ *M*^−1^*v*/2, which are commonly referred to as the potential energy and kinetic energy respectively. The total Hamiltonian energy function is *H*(*β*, *v*) = *U*(*β*) + *K*(*v*).

At each state (*β*, *v*), a new proposal (β*, v*) is generated by simulating Hamiltonian dynamics satisfying the Hamilton’s equations:

$$\frac{\partial \beta }{\partial t}=\frac{\partial H(\beta ,v)}{\partial v}=M^{-1}v;\;\frac{\partial v}{\partial t}=-\frac{\partial H(\beta ,v)}{\partial \beta }=\frac{\partial \text{ log }\Pi (\beta |D)}{\partial \beta }.$$

Since the exact solution to the above is intractable, we can numerically approximate the temporal evolution using the leapfrog scheme, as described in the following pseudocode.


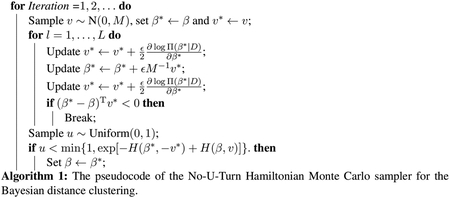


To accelerate the convergence of the Markov chain to stationarity, we first use the BFGS optimization algorithm (implemented in the PyTorch package) to first minimize *U*(*β*) and obtain the posterior mode $\hat{\beta }$. We then initialize the Markov chain at $\beta =\hat{\beta }$.

A typical choice for the working parameter *M*^*−*1^ is to let it roughly scale with the covariance matrix of the posterior distribution (Neal, 2011). Using $\hat{\beta }$, we calculate the observed Fisher information at $\hat{\beta }$ [the Hessian matrix of *U*(*β*) evaluated at $\hat{\beta }$, denoted by ${\text{Hess}}_{U}(\hat{\beta })$], which gives an approximation to the inverse covariance of *β*. Although it is tempting to set $M^{-1}={\left[{\text{Hess}}_{U}(\hat{\beta })\right]}^{-1}$, the matrix inversion of the latter is often costly and ill-conditioned. To avoid this problem, we use a simpler and diagonal parameterization $M^{-1}=\text{diag}\left(1/{\text{Hess}}_{U}{\left(\hat{\beta }\right)}_{i,i}\right)$, which shows good empirical performances in all the examples within this article.

To run the HMC sampler, we use the No-U-Turn Sampler (NUTS-HMC) algorithm (Hoffman and Gelman, 2014) implemented in the ‘hamiltorch’ package (Cobb and Jalaian, 2020), which also automatically tunes the other two working parameters *ϵ* and *L*. After the automatic tuning, the algorithm reaches an acceptance rate close to 70% as commonly desired for good mixing of the Markov chains. To provide some running time, using a quad-core i7 CPU, at *n* = 1000, the HMC algorithm takes about 20 minutes for running 10, 000 iterations.

**Remark 11** *On the computational cost*, *the most expensive step in the HMC algorithm is the calculation of the derivative of* log *G*(*D*; *W*) *with respect to the matrix W*, *which involves the following form:*

$$\frac{\partial \text{tr}\left[\left(X^{\text{T}}\text{AX}\right){\left(X^{\text{T}}\text{BX}\right)}^{-1}\right]}{\partial X}=2\text{AX}{\left(X^{\text{T}}\text{BX}\right)}^{-1}-2\text{BX}{\left(X^{\text{T}}\text{BX}\right)}^{-1}\left(X^{\text{T}}\text{AX}\right){\left(X^{\text{T}}\text{BX}\right)}^{-1}$$

*where $X\in {\mathbb{R}}^{n\times k}$*, *symmetric $B\in {\mathbb{R}}^{n\times n}$ and symmetric $A\in {\mathbb{R}}^{n\times n}$. Since k is relatively small*, *the matrix inversion of the k*×*k matrix is not costly $\left[O\left(k^{3}\right)\right]$ and dominated by the matrix multiplication $O\left(n^{2}k\right)$. Therefore*, *running over L leapfrog steps*, *the computational cost per iteration of HMC is $O\left(Ln^{2}k\right)$*.

*Potentially*, *one could instead consider a Gibbs sampling algorithm*, *using a block*-*wise update of C*^T^(log *D*)*C and C*^T^*DC (instead of a full evaluation of the matrix product) when sampling each row of C. Despite having a similar computing complexity*, *a strength of HMC is that we can take advantage of the highly parallelized matrix operation on C*, *which is often faster than the sequential looping over each row of C*.

*In comparison*, *the parametric*/*model*-*based clustering algorithm has a lower cost of O*(*n*), *although this often comes with a risk of model misspecification for modern data. Therefore*, *choosing which class of methods involves a trade*-*off between computational speed versus model robustness*.

The posterior samples of *CC*^T^ give an estimate of the pairwise co-assignment probabilities $\text{pr}\left(c_{i}=c_{j}\right)=\sum_{h=1}^{k}\text{pr}\left(c_{i}=c_{j}=h\right)$. To obtain estimates for pr(*c*_*i*_ = *h*), we use symmetric simplex matrix factorization (Duan, 2020) on {pr(*c*_*i*_ = *c*_*j*_)}_*i,j*_ to obtain an *n* × *k* matrix corresponding to {pr(*c*_*i*_ = *h*)}_*i,h*_. For the diagnostics on the convergence, we calculate the autocorrelation (ACF) and the effective sample size (ESS) for each parameter, and we provide some diagnostic plots in the appendix.

In this article, for the ease of visualization and interpretation, we use $\text{pr}\left(c_{i}\neq {\hat{c}}_{i}|D\right)$ as a measure of the uncertainty on the point estimate ${\hat{c}}_{i}={\text{max}}_{h=1,\ldots ,k}\text{ pr}\left(c_{i}=h|D\right)$. An alternative is to use the variation of information (Wade and Ghahramani, 2018) as a metric between the clusterings, leading to the discrete extension of the posterior mean and credible intervals. The readers can find the method and toolbox within the reference.

In addition, in the appendix, we show that the non-negative matrix factorization (NMF) algorithm, if using a calibrated similarity matrix as its input (such as using our distance likelihood), produces an almost indistinguishable result from the Bayesian distance clustering method. On the other hand, if the similarity is set less carefully (such as using the “default” choice in popular machine learning packages), we found a severe sensitivity that leads to over-/under-estimation of the uncertainty (as shown in Panel 4 of Figure 8). Therefore, for the sake of both conciseness and fairness, we choose to not present the NMF results without calibration in the numerical experiments.

## 6. Numerical experiments

### 6.1 Clustering with skewness-robust distance

As described in Section 2.1, the vector norm-based distance is automatically robust to skewness. To illustrate, we generate *n* = 200 data from a two-component mixture of skewed Gaussians:

$$\text{pr}\left(c_{i}=1\right)=\text{pr}\left(c_{i}=2\right)=0.5,\;$$

$$y_{i,j}|c_{i}=h\sim \text{SN}\left(\mu_{h},1,\alpha_{h}\right)\text{ for }j=1\ldots p,$$

where SN(*μ*, *σ*, *α*) has density *π*(*y* | *μ*, *σ*, *α*) = 2*f*{(*y* − *μ*)/*σ*}*F*{*α*(*y* − *μ*)/*σ*}with *f* and *F* the density and cumulative distribution functions for the standard Gaussian distribution.

**Figure 3:**
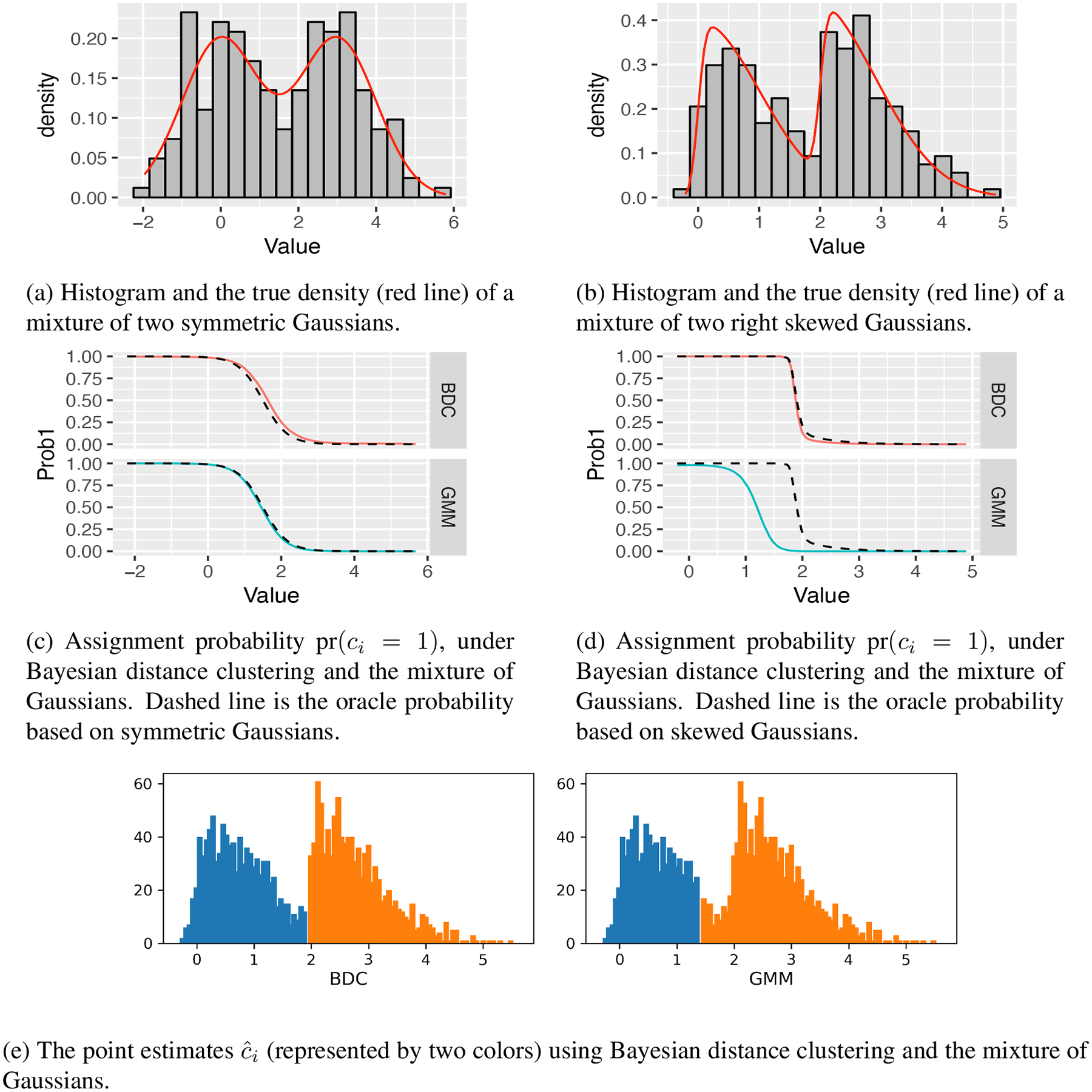
Clustering data from a two-component mixture of skewed Gaussians in ℝ. Bayesian Distance clustering (BDC) gives posterior clustering probabilities close to the oracle probabilities regardless of whether the distribution is skewed or not (upper plots in panel c and d), while the mixture of Gaussians fails when the skewness is present (lower plot in panel d).

We start with *p* = 1 and assess the performance of the Bayesian distance clustering model under both non-skewed (*α*_1_ = *α*_2_ = 0, *μ*_1_ = 0, *μ*_2_ = 3) and skewed distributions (*α*_1_ = 8, *α*_2_ = 10, *μ*_1_ = 0, *μ*_2_ = 2). The results are compared against the mixture of Gaussians as implemented in the *Mclust* package. Figure 3(a,c) show that for non-skewed Gaussians, the proposed approach produces clustering probabilities close to their oracle probabilities, obtained using knowledge of the true kernels that generated the data. When the true kernels are skewed Gaussians, Figure 3(b,d) shows that the mixture of Gaussians gives inaccurate estimates of the clustering probability, whereas Bayesian distance clustering remains similar to the oracle.

To evaluate the accuracy of the point estimate ${\hat{c}}_{i}$, we compute the adjusted Rand index (Rand, 1971) with respect to the true labels. We test under different *p* ∈ {1, 5, 10, 30}, and repeat each experiment 30 times. The results are compared against model-based clustering using symmetric and skewed Gaussians kernels, using independent variance structure. As shown in Table 1, the misspecified symmetric model deteriorates quickly as *p* increases. In contrast, Bayesian distance clustering maintains high clustering accuracy.

### 6.2 Clustering high dimensional data with subspace distance

For high-dimensional clustering, it is often useful to impose the additional assumption that each cluster lives near a different low-dimensional manifold. Clustering data based on these manifolds is known as subspace clustering. We exploit the sparse subspace embedding algorithm proposed by Vidal (2011) to learn pairwise subspace distances. Briefly speaking, since the data in the same cluster are alike, each data point can be approximated as a linear combination of several other data points in the same subspace; hence a sparse locally linear embedding can be used to estimate an *n* × *n* coefficient matrix $\hat{W}$ through

$$\hat{W}=\text{arg }\underset{W:w_{i,i}=0,\sum_{j}w_{i,j}=1}{\text{min}}\sum_{i=1}^{n}{\left\|y_{i}-Wy_{i}\right\|}_{2}^{2}+\|W{\|}_{1},$$

where the sparsity of $\hat{W}$ ensures only the data in the same linear subspace can have non-zero embedding coefficients. Afterward, we can define a subspace distance matrix as

$$d_{i,j}=2-\left(\frac{\left|{\hat{w}}_{i,j}\right|}{{\text{max}}_{j^{\prime }}\left|{\hat{w}}_{i,j^{\prime }}\right|}+\frac{\left|{\hat{w}}_{j,i}\right|}{{\text{max}}_{j}\left|{\hat{w}}_{j,j}\right|}\right),$$

where we follow Vidal (2011) to normalize each row by its absolute maximum. We then use this distance matrix in our Bayesian distance clustering method.

To assess the performance, we use the MNIST data of hand-written digits of 0 – 9, with each image having *p* = 28 × 28 pixels. In each experiment, we take *n* = 10, 000 random samples to fit the clustering models, among which each digit has approximately 1000 samples, and we repeat experiments 10 times. For comparison, we also run the near low-rank mixture model in *HDclassif* package (Bergé et al., 2012) and spectral clustering based on the *p*-dimensional vector norm. Our method using subspace distances shows clearly higher accuracy, as shown in Table 2.

## 7. Clustering brain regions

We carry out a data application to segment the mouse brain according to the gene expression obtained from the Allen Mouse Brain Atlas dataset (Lein et al., 2007). Specifically, the data are *in situ* hybridization gene expression, represented by expression volume over spatial voxels. Each voxel is a (200*μm*)^3^ cube. We take the mid-coronal section of 41 × 58 voxels. Excluding the empty ones outside the brain, they have a sample size of *n* = 1781. For each voxel, there are records of expression volume over 3241 different genes. To avoid the curse of dimensionality for distances, we extract the first *p* = 30 principal components and use them as the source data.

Since gene expression is closely related to the functionality of the brain, we will use the clusters to represent the functional partitioning, and compare them in an unsupervised manner with known anatomical regions. The voxels belong to 12 macroscopic anatomical regions (Table 4).

For clustering, we use an over-fitted mixture with *k* = 20 and small Dirichlet concentration parameter *α* = 1/20. As shown by Rousseau and Mengersen (2011), asymptotically, small *α* < 1 leads to the automatic emptying of small clusters; we observe such behavior here in this large sample. In the Markov chain, most iterations have 7 major clusters. Table 5 lists the voxel counts at ${\hat{c}}_{\left(n\right)}$.

**Figure 5:**
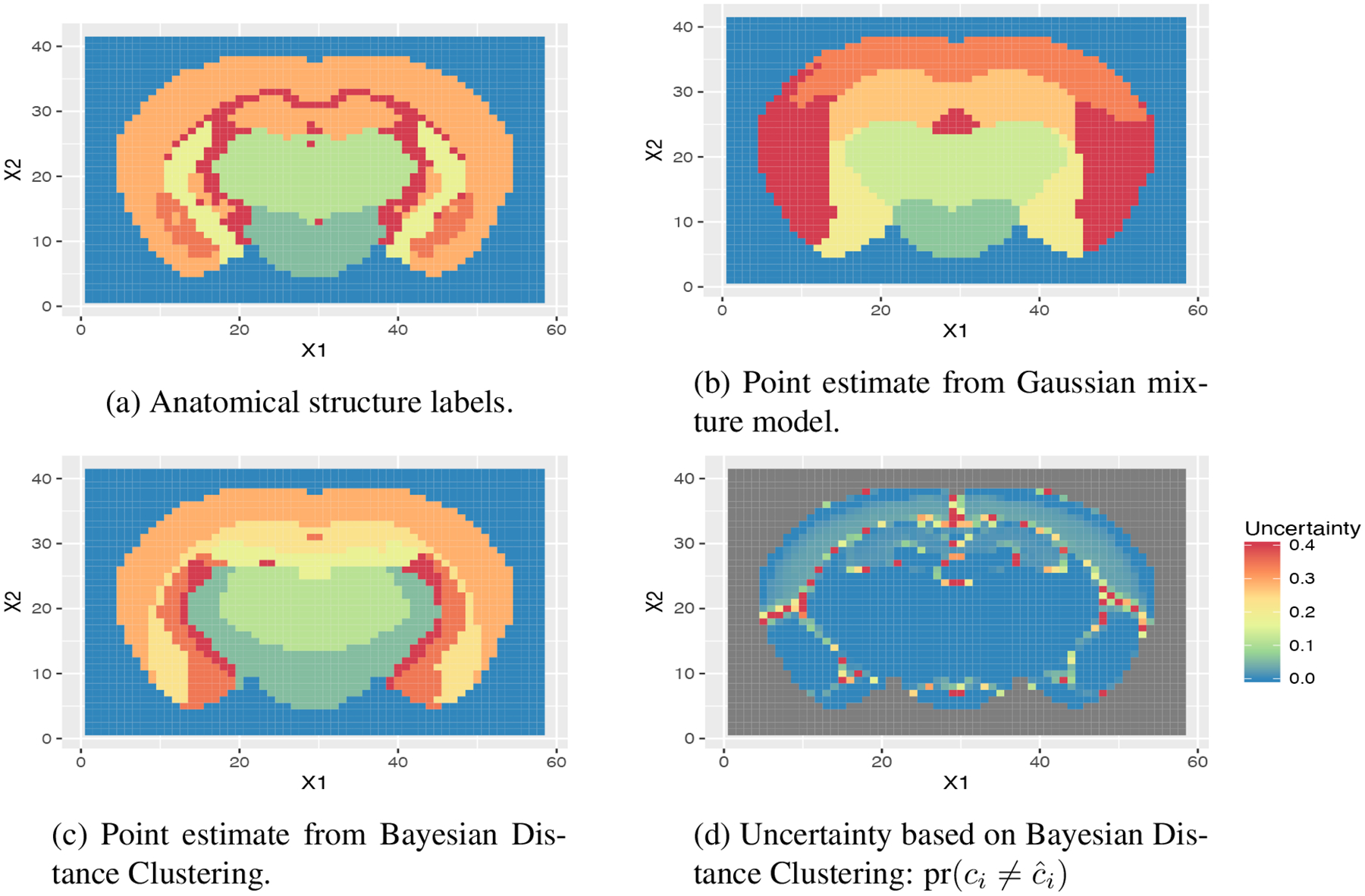
Clustering mouse brain using gene expression: visualizing the clustering result on the spatial grid of brain voxels. Comparing with the anatomical structure (panel a), Bayesian Distance Clustering (panel c) has a higher similarity than the Gaussian mixture model (panel b). Most of the uncertainty (panel d) resides in the inner layers of the cortical plate (upper parts of the brain).

**Figure 8:**
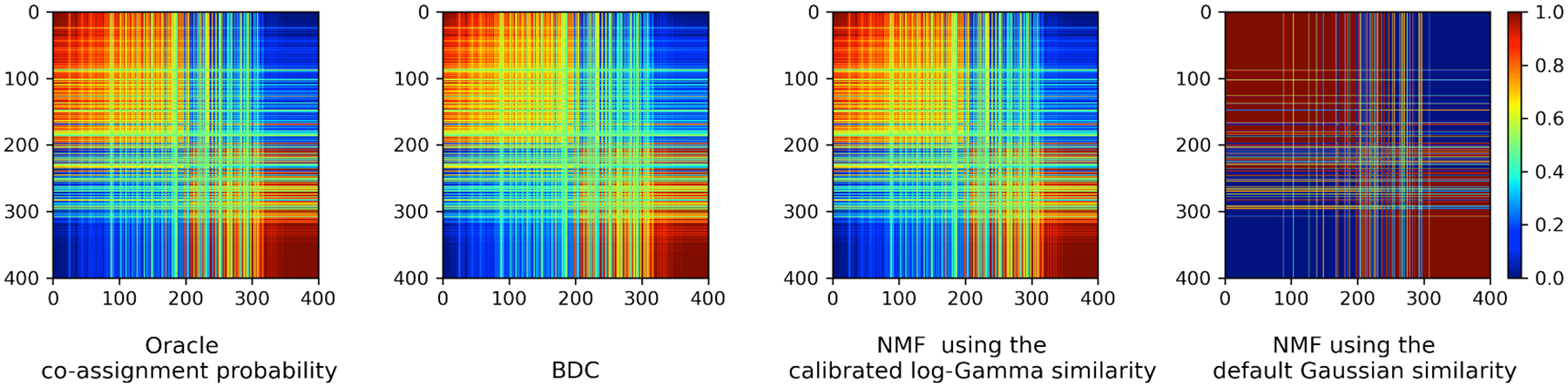
Comparing the performance of the Bayesian distance clustering (BDC) and the nonnegative matrix factorization (NMF) using the symmetric simplex matrix factorization (Zhao et al., 2015; Duan, 2020). We apply the algorithms on the two clusters of skew Gaussian data as generated in Section 2.1, and plot the estimated co-assignment probability matrix for each method. As can be seen from Panels 2 and 3, when NMF is well calibrated in its similarity function (as we defined in (13), with parameters set to the posterior mean estimate from BDC), it produces a result almost indistinguishable from BDC — both are very close to the oracle co-assignment probability (Panel 1). On the other hand, NMF using an uncalibrated similarity $\text{exp}\left(-d_{i,j}^{2}\right)$ produces a result (Panel 4) very different from the oracle.

Comparing the two tables, although we do not expect a perfect match between the structural and functional partitions, we do see a correlation in group sizes based on the top few groups. Indeed, visualized on the spatial grid (Figure 5), the point estimates from Bayesian distance clustering have very high resemblance to the anatomical structure. Comparatively, the clustering result from the Gaussian mixture model is completely different.

To benchmark against other distance clustering approaches, we compute various similarity scores and list the results in Table 3. Competing methods include spectral clustering (Ng et al., 2002), DBSCAN (Ester et al., 1996) and HDClassif (Bergé et al., 2012); the first two are applied on the same dimension-reduced data as used by Bayesian distance clustering, while the last one is applied directly on the high dimensional data. Among all the methods, the point estimates of Bayesian Distance Clustering have the highest similarity to the anatomical structure.

Figure 5(d) shows the uncertainty about the point clustering estimates, in terms of the probability $\text{pr}\left(c_{i}\neq {\hat{c}}_{i}\right)$. Besides the area connecting neighboring regions, most of the uncertainty resides in the inner layers of the cortical plate (upper parts of the brain). As a result, the inner cortical plate can be either clustered with the outer layer or with the inner striatum region. Therefore, from a practical perspective, when segmenting the brain according to the functionality, it is more appropriate to treat the inner layers as a separate region.

## 8. Discussion

The use of a distance likelihood reduces the sensitivity to the choice of a mixture kernel, giving the ability to exploit distances for characterizing complex and structured data. While we avoid specifying the kernel, one potential weakness is that there can be sensitivity to the choice of the distance metrics. For example, the Euclidean distance tends to produce a more spherical cluster, compared to the weighted Euclidean distance (see appendix). However, our results suggest that this sensitivity is often less than that of the assumed kernel. In many settings, there is a rich literature considering how to carefully choose the distance metric to reflect structure in the data (Pandit and Gupta, 2011). In such cases, the sensitivity of clustering results to the distance can be viewed as a positive. Clustering method necessarily relies on some notion of distances between data points.

Another issue is that we give up the ability to characterize the distribution of the original data. An interesting solution is to consider a modular modeling strategy that connects the distance clustering to a post-clustering inference model while restricting the propagation of cluster information in one direction only. Related modular approaches have been shown to be much more robust than a single overarching full model (Jacob et al., 2017).

Our concentration characterization of the within-cluster distance based on the vector norm holds for any arbitrary *p*. On the other hand, high-dimensional clustering is a subtle topic with challenging issues: (i) not all the coordinates in ${\mathbb{R}}^{p}$ contain discriminative information that is favorable for one particular partition; hence some alternative distances (Vidal, 2011), feature selection (Witten and Tibshirani, 2010), or multi-view clustering (Duan, 2020) may be necessary; (ii) the selection of number of clusters *k* becomes difficult, and it was recently discovered (Chandra et al., 2020) that the model-based framework could lead to nonsensical results of converging to either one cluster or *n* clusters even under a correctly specified model, as *p* → ∞

One interesting extension of this work is to combine with the nearest neighbor algorithm — we can choose to ignore the large distances and instead focus on modeling the *K* smallest ones for each data point — this could also significantly reduce the $O\left(n^{2}\right)$ computing and storage cost, via the sparse matrix computation. One possible model is to replace our Gamma distance density with a two-component mixture: one Gamma component concentrating near zero for modeling small distances, and one Uniform(0, max_*i,j*_ *d*_*i,j*_) for handling large distances. Since the uniform density is a constant that does not depend on the specific value of the distance (as long it is in the support of the uniform), it effectively eliminates the influence of large distances in clustering.

## Figures and Tables

**Figure 2:**
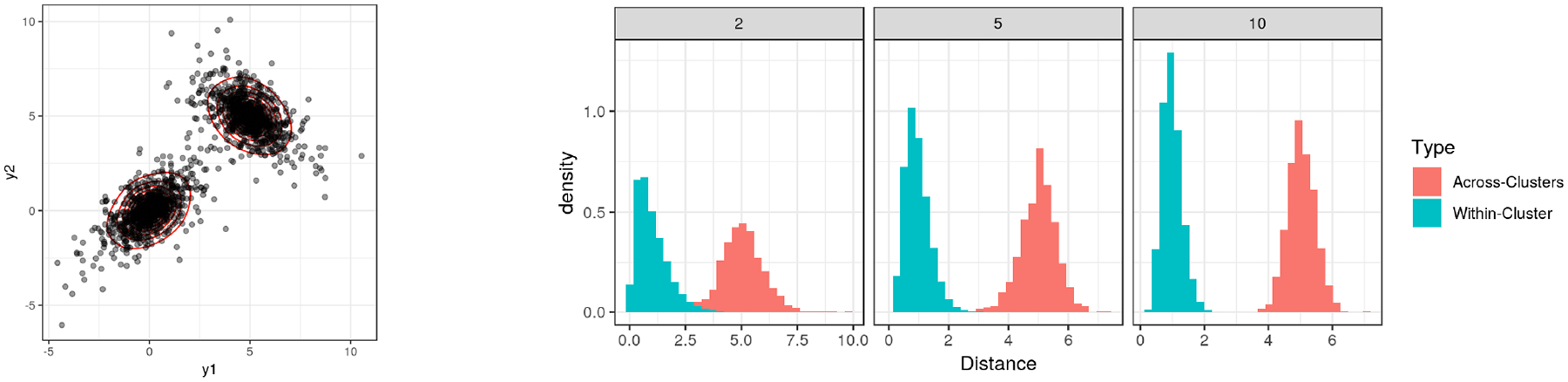
Histograms of Euclidean distances scaled by 1/*σ*_*h*_ (with $\sigma_{h}\approx \sqrt{p}$). Left is the first two dimensions, and the right show that the distances formed within a cluster (cyan) tend to be much smaller than the ones across clusters (red). Each cluster’s data are generated from a multivariate Laplace distribution $y_{i}\sim \text{Lap}\left(\mu_{h},\Sigma_{h}\right)$ with *h* = 1, 2.

**Figure 4:**
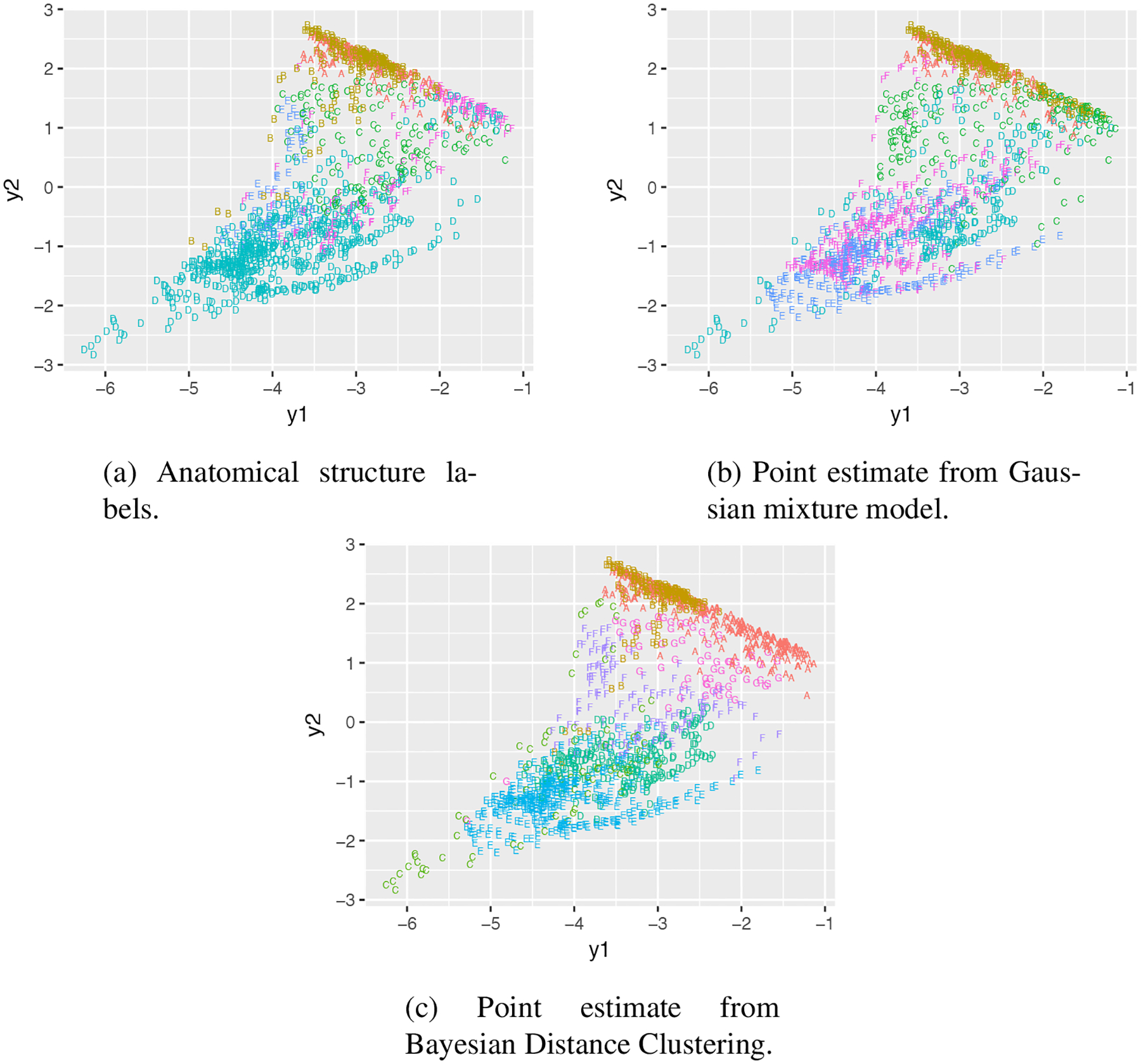
Clustering mouse brain using gene expression: visualizing the clustering result on the first two principal components.

**Table 1:** Accuracy of clustering skewed Gaussians under different dimensions *p*. Adjusted Rand index (ARI) is computed for the point estimates using variation of information. The average and 95% confidence interval are shown.

*p*	Bayes Dist. Clustering	Mix. of Gaussians	Mix. of Skewed Gaussians
1	0.80 (0.75, 0.84)	0.65 (0.55, 0.71)	0.81 (0.75, 0.85)
5	0.76 (0.71, 0.81)	0.55 (0.40, 0.61)	0.76 (0.72, 0.80)
10	0.72 (0.68, 0.76)	0.33(0.25, 0.46)	0.62 (0.53, 0.71)
30	0.71 (0.67, 0.76)	0.25 (0.20, 0.30)	0.43 (0.37, 0.50)

**Table 2:** Accuracy of clustering MNIST hand-written digit data. Adjusted Rand index (ARI) is computed for the point estimates using variation of information. The average ARI and 95% confidence intervals are shown.

Bayes Dist. Clustering	Spectral Clustering	HDClassif
0.57 (0.54, 0.60)	0.50 (0.48, 0.52)	0.35 (0.31, 0.43)

**Table 3:** Comparison of label point estimates using Bayesian distance clustering (BDC), Gaussian mixture model (GMM), spectral clustering, DBSCAN and HDClassif. The similarity measure is computed with respect to the anatomical structure labels.

	BDC	GMM	Spectral Clustering	DBSCAN	HDClassif
Adjusted Rand Index	0.49	0.31	0.45	0.43	0.43
Normalized Mutual Information	0.51	0.42	0.46	0.44	0.47
Adjusted Mutual Information	0.51	0.42	0.47	0.45	0.47

**Figure 6:**
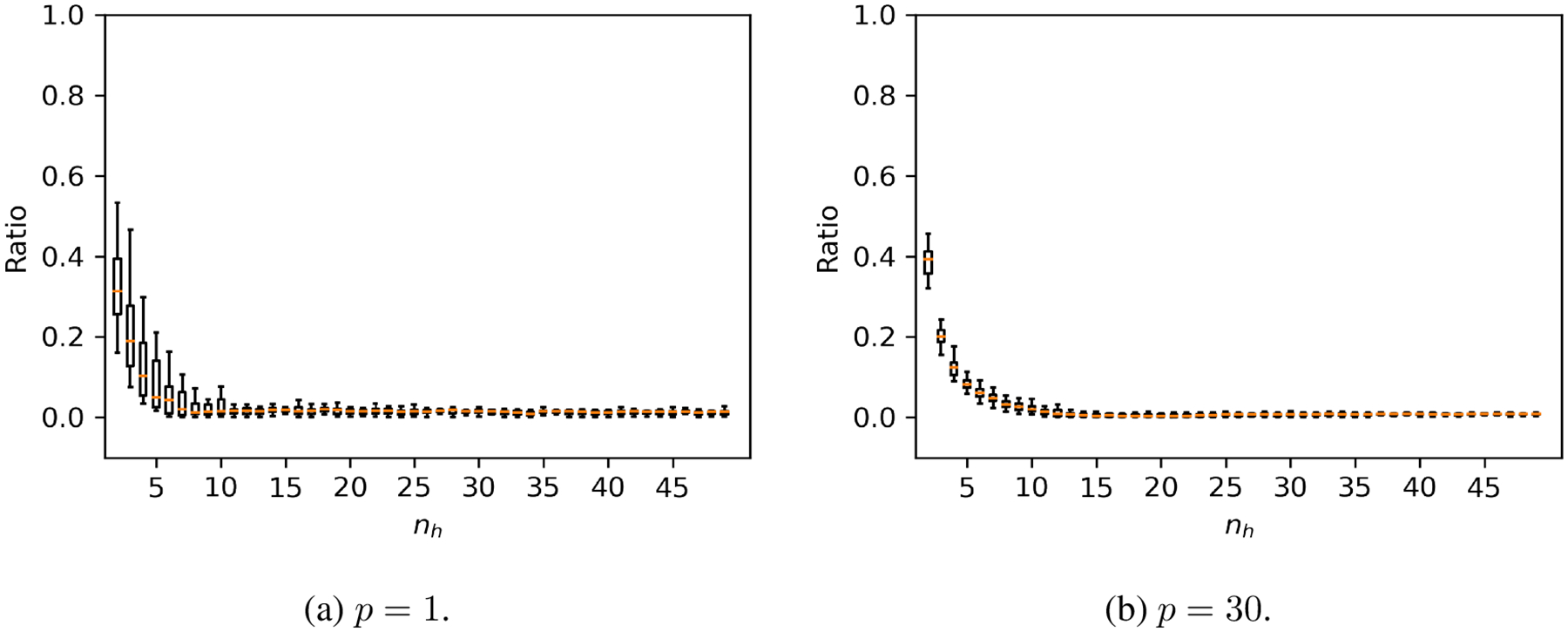
Numerical experiments show that the seed conditional density $K_{h}\left(y_{1}^{\left[h\right]}|{\tilde{d}}_{2,1}^{\left[h\right]},\ldots ,{\tilde{d}}_{n_{h},1}^{\left[h\right]}\right)$ becomes much smaller than the distance likelihood as *n*_*h*_ increase.

**Figure 7:**
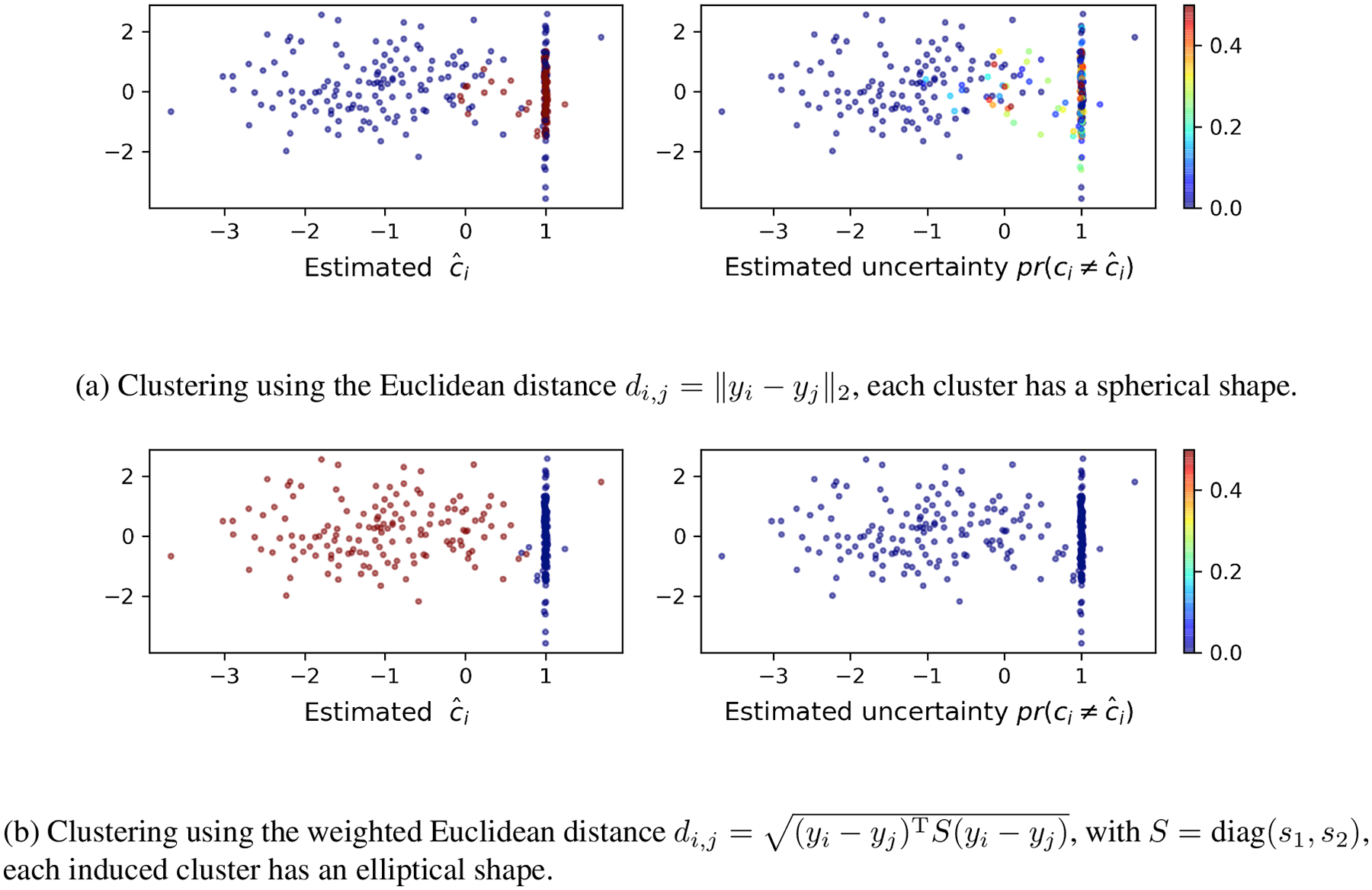
Experiments show that the choice of distances may impact the shapes of the clusters and the uncertainty. Although, if one wants to separate clusters based on different ‘shapes’, a model on the cluster-specific covariances could be more useful than one on the pairwise distances.

## Appendix

### Proof of Lemma 2

**Proof** We first focus on *x* ~ Gamma(*α*, 1), by the Markov’s inequality

$$\text{pr}(x\geq t)\leq \frac{E\text{ exp}(\text{sX})}{\text{exp}(\text{st})}={\left(1-s\right)}^{-\alpha }e^{-ts},$$

where *s* < 1. Minimizing the right hand side over *s* yields *s** = 1 −*α*/*t*, and

$$\text{pr}(x\geq t)\leq {\left(\frac{t}{\alpha }\right)}^{\alpha }e^{-t+\alpha }=\alpha^{-\alpha }e^{\alpha }t^{\alpha }e^{-t}.$$

Scaling *x* by *σ*_*h*_ and adjusting the constant yield the results. ■

### Proof of Lemma 4

**Proof** Equivalently, the sub-exponential tail can be characterized by the bound on its moment generating function

$$E\text{ exp}\left\{t\left(y_{i,k}^{\left[h\right]}-\mu_{k}^{\left[h\right]}\right)\right\}\leq \text{exp}\left(\nu_{h}^{2}t^{2}/2\right)\;\forall |t|\leq 1/b_{h},$$

for *k* = 1, …, *p*. It immediately follows that the pairwise difference ${\tilde{d}}_{i,j}^{\left[h\right]}=y_{i}^{\left[h\right]}-y_{j}^{\left[h\right]}$ between two iid random variables must be sub-exponential as well, with

$$E\text{ exp}\left(t{\tilde{d}}_{i,j,k}^{\left[h\right]}\right)\leq \text{exp}\left(\nu_{h}^{2}t^{2}\right)\;\forall |t|\leq 1/b_{h}.$$

That is, sub-exponential $-\left(\sqrt{2}\nu_{h},b_{h}\right)$. Then the vector norm

$$\text{pr}\left(d_{i,j}^{\left[h\right]}>p^{\eta }t\right)=\text{pr}\left(\sum_{k=1}^{p}{\left|{\tilde{d}}_{i,j,k}^{\left[h\right]}\right|}^{q}>p^{\eta q}t^{q}\right)\;\leq p\text{ pr}\left({\left|{\tilde{d}}_{i,j,k}^{\left[h\right]}\right|}^{q}>p^{\eta q-1}t^{q}\right)\;=p\text{ pr}\left(\left|{\tilde{d}}_{i,j,k}^{\left[h\right]}\right|>p^{\eta -1/q}t\right)\;\leq 2p\text{ exp}\left\{-tp^{\left(\eta -1/q\right)}/\left(2b_{h}\right)\right\}\text{ for }t>p^{1/q-\eta }2\nu_{h}^{2}/b_{h}.$$

where the first inequality is due to $\text{pr}\left(\sum_{i=1}^{p}a_{i}>b\right)\leq \text{pr}\left(\text{There is at least one }i:a_{i}>b/p\right)\leq \sum_{i=1}^{p}\text{pr}\left(a_{i}>b/p\right)$ and second inequality uses $Ed_{i,j,k}^{\left[h\right]}=0$ and the property of sub-exponential tail (Wainwright, 2019). ■

### Proof of Lemma 7

**Proof** For a clear exposition, we omit the sub/super-script *h* for now and use *x*_*i*_ = *T*(*y*_*i*_)

$$E_{y_{i}}E_{y_{j}}\sum_{i=1}^{n}\sum_{j=1}^{n}B_{\phi }\left(x_{i},x_{j}\right)=E_{y_{i}}E_{y_{j}}\sum_{i=1}^{n}\sum_{j=1}^{n}\left\{\phi \left(x_{i}\right)-\phi \left(x_{j}\right)-\langle x_{i}-x_{j},\nabla \phi \left(x_{j}\right)\rangle \right\}\;=E_{y_{j}}\sum_{j=1}^{n}\sum_{i=1}^{n}\{E_{y_{i}}\phi \left(x_{i}\right)-\phi (\mu )-\langle E_{y_{i}}x_{i}-\mu ,\nabla \phi (\mu )\rangle \;+\phi (\mu )-\phi \left(x_{j}\right)-\langle E_{y_{i}}x_{i}-x_{j},\nabla \phi \left(x_{j}\right)\rangle \;=n\sum_{i=1}^{n}E_{y_{i}}\left\{\phi \left(x_{i}\right)-\phi (\mu )-\langle x_{i}-\mu ,\nabla \phi (\mu )\rangle \right\}\;+n\sum_{j=1}^{n}E_{y_{j}}\left\{\phi (\mu )-\phi \left(x_{j}\right)-\langle \mu -x_{j},\nabla \phi \left(x_{j}\right)\rangle \right\}\;=n\sum_{i=1}^{n}E_{y}\left\{B_{\phi }\left(x_{i},\mu \right)+B_{\phi }\left(\mu ,x_{i}\right)\right\},$$

where 〈., .〉 denotes dot product, the second equality is due to Fubini theorem and $E_{y_{i}}x_{i}-\mu =0$. ■

### Proof of Lemma 9

**Proof** Using **1**_*n,m*_ *n* × *m* matrix with all elements equal 1. Since *C*^*T*^*C* = *diag*(*n*_1_, …, *n*_*k*_), the 2 times of normalized graph cut loss can be written as

$$\text{tr}\left[A\left(1_{n,k}-C\right){\left(C^{\text{T}}C\right)}^{-1}C^{\text{T}}\right]\;=-\text{tr}\left\{AC{\left(C^{\text{T}}C\right)}^{-1}C^{\text{T}}\right\}+\text{tr}\left[A1_{n,k}{\left(C^{\text{T}}C\right)}^{-1}C^{\text{T}}\right].$$

For the second term

$$\text{tr}\left[A1_{n,k}{\left(C^{\text{T}}C\right)}^{-1}C^{\text{T}}\right]\;=\text{tr}\left[C^{\text{T}}A1_{n,k}{\left(C^{\text{T}}C\right)}^{-1}\right]\;=\sum_{i=1}^{n}\frac{\sum_{j=1}^{n}A_{i,j}}{n_{c_{i}}}.$$

■

**Table 4:** Names and voxel counts in 12 macroscopic anatomical structures in the coronal section of the mouse brain. They represent the *structural* partitioning of the brain.

Anatomical Structure Name	Voxel Count
Cortical plate	718
Striatum	332
Thalamus	295
Midbrain	229
Basic cell groups and regions	96
Pons	56
Vermal regions	22
Pallidum	14
Cortical subplate	6
Hemispheric regions	6
Cerebellum	5
Cerebral cortex	2

**Table 5:** Group indices and voxel counts in 7 clusters found by Bayesian Distance Clustering, using the gene expression volume over the coronal section of the mouse brain. They represent the *functional* partitioning of the brain.

Index	Voxel Count
1	626
2	373
3	176
4	113
5	79
6	39
7	12

### Numerical experiments showing the effect of discarding the seed

We show numerically that as *n*_*h*_ increases, the seed conditional density $K_{h}\left(y_{1}^{\left[h\right]}|{\tilde{d}}_{2,1}^{\left[h\right]},\ldots ,{\tilde{d}}_{n_{h},1}^{\left[h\right]}\right)$ becomes very small in magnitude compared to the distance likelihood $G_{h}\left({\tilde{d}}_{2,1}^{\left[h\right]},\ldots ,{\tilde{d}}_{n_{h},1}^{\left[h\right]}\right)$. To compute those two terms, we use the Gaussian example presented in Section 2.1, simulated with $\sigma_{h}^{2}=1$, *μ*_*h*_ = 0. Since the values of those densities are very close to zero, we use the log-scale and compute the ratio,

$$\left|\frac{\text{log }K_{h}\left(y_{1}^{\left[h\right]}|{\tilde{d}}_{2,1}^{\left[h\right]},\ldots ,{\tilde{d}}_{n_{h},1}^{\left[h\right]}\right)}{\text{log }K_{h}\left(y_{1}^{\left[h\right]}|{\tilde{d}}_{2,1}^{\left[h\right]},\ldots ,{\tilde{d}}_{n_{h},1}^{\left[h\right]}\right)G_{h}\left({\tilde{d}}_{2,1}^{\left[h\right]},\ldots ,{\tilde{d}}_{n_{h},1}^{\left[h\right]}\right)}\right|$$

as a measure of how small $K_{h}\left(y_{1}^{\left[h\right]}|.\right)$ compared to *G*_*h*_. We repeat each experiment for 30 times and create the box plots. As can be seen from Figure 6 (left panel), the ratio quickly drops to near zero after *n*_*h*_ ≥10. In addition, we repeat the same experiment except using a multivariate Gaussian N(0, *I*_*p*_); and the findings are very similar (right panel).
